# Effects of Vertical Integration Reform on Primary Healthcare Institutions in China: Evidence From a Longitudinal Study

**DOI:** 10.34172/ijhpm.2021.93

**Published:** 2021-08-21

**Authors:** Shasha Yuan, Fengmei Fan, Dawei Zhu

**Affiliations:** ^1^Institute of Medical Information & Library, Chinese Academy of Medical Sciences & Peking Union Medical College, Beijing, China.; ^2^Beijing Huilongguan Hospital, Peking University Huilongguan Clinical Medical School, Beijing, China.; ^3^China Center for Health Development Studies, Peking University, Beijing, China.

**Keywords:** Vertical Integration, Integrated Care, Primary Healthcare, Policy Effect, China

## Abstract

**Background:** Integrated care is a global trend in international healthcare reform, particularly for piloting vertical integration involving hospitals and primary healthcare institutions (PHIs). However, evidence regarding the impact of vertical integration on primary healthcare has been mixed and limited. Our study aims to evaluate the empirical effects of vertical integration reform on PHIs in China, and examines variations across integration intensity (tight integration vs. loose collaboration).

**Methods:** This study used a longitudinal design. The time-varying difference-in-difference (DID) method with a fixed-effect model for panel data was adopted. A total of 370 PHIs in the eastern, central, and western areas of China from 2009 to 2018 were covered. Outcome measures included the indicators at three dimensions regarding inpatient and outpatient service volume, patient flow between PHIs and hospitals and quality of chronic disease care (hypertension and diabetes).

**Results:** Significant increases in absolute (the number) and relative (the ratio between PHIs and hospitals) volume of inpatient admissions have been found after reform under tight integration, peaking at 183% and 15.0% respectively, in the third reform year. The quality of hypertension and diabetes care (by indicators of control rate of blood pressure and blood glucose) showed significant improvements under both types of vertical integration after reform. It was much more distinct for the PHIs under tight integration, which had the most significant increase of 34.0% and 22.8% under tight integration for the control rate of hypertension and diabetes compared to the peak of 21.2% and 22.1% respectively under loose collaboration.

**Conclusion:** Our findings suggest that vertical integration (especially tight integration) in China significantly contributed to strengthening primary healthcare in terms of inpatient services and quality of hypertension and diabetes care, providing empirical evidence to other countries on integrating primary healthcare-based health systems.

## Background

**Key Messages**

** Implications for policy makers**

- This study examined the effects of vertical integration reform on primary healthcare institutions (PHIs) in China, providing significant empirical evidence from developing countries and providing valuable guidance to strengthen integrated primary care-based health systems in practice.
- When designing vertical integration reform, tight integration between PHIs and hospitals can be maintained. Our study demonstrated significantly positive effects of tight integration on the inpatient service volume, inpatient flow, and quality of hypertension and diabetes care.
- Along with the vertical integration reform, priority strategies are suggested, including strengthening leading hospitals’ operation and management responsibilities at the administrative level, establishing or rebuilding departments with high demands, more coordination at PHIs (such as rehabilitation beds) at the organizational level, and providing regular professional training to primary care workers at the service delivery level. These reasons were most likely to have stimulated the positive effects of vertical integration reform in China.

** Implications for the public**

Our study explored vertical integration piloting experiences and their effects on primary healthcare institutions (PHIs) in China. Our analysis revealed that both types of vertical integration had a significantly positive effect on quality of hypertension care and diabetes care; tight integration had significantly increased the number of inpatient admissions at PHIs and inpatient flow between PHIs and hospitals while no significant effects were observed for these indicators at the PHIs after loose collaboration. Given the positive effects observed, our study suggests that vertical integration (especially tight integration) in China significantly contributed to strengthening primary healthcare, providing empirical evidence for other countries in establishing integrated primary healthcare-based health systems.

Integrated care is a global trend in international healthcare reform, which has attracted considerable attention as an essential path to developing better and more cost-effective healthcare systems.^1^ However, owing to its multiple dimensions and various scopes, it is challenging to define a uniform concept of integration.^2^ Studies concerning the impacts of integration differ across settings and countries. For example, in high-income countries, some evidence indicates that integrated care models produce better patient experience but play a small role in improving health outcomes.^3-5^ The findings on cost-effectiveness are also contradictory, when integrated care experiences and outcomes in different countries are compared.^4^ Notably, huge variations are found in the integration forms, approaches, and scopes between high-income countries and low-and middle-income countries. A comprehensive review conducted by Mounier-Jack et al showed that evidence on integrated care outcomes, particularly from low-and middle-income countries, was scant.^2^

 A process-based definition widely accepted by many national governments points out, “Integration is a coherent set of methods and models on the funding, administrative, organizational, service delivery and clinical levels designed to create connectivity and collaboration within and between cure and care sectors.”^1,6^ Several integrated care models and taxonomies have been developed.^7^ Among these, vertical integration has received more attention in practice which includes coordination of care across primary, community, and hospital care by bringing together health organizations at different levels under one management model.^6^ The opposite form is horizontal integration, which typically involves organizations at the same stage in delivering care.^6,8^ Combined with ongoing integration reform in China, this study mainly focused on vertical integration within the healthcare system.

 In China, the fragmentation of primary healthcare and hospital care has been frequently criticized. Specifically, primary healthcare institutions (PHIs) in this study refer to township health centers (THCs) in rural areas and community health centers (CHCs) in urban areas,^9^ which are usually set at the town (rural area) or street (urban area) level. The functions of PHIs mainly include providing generalist care and public health services, and they are funded by the government.^10^ However, only a portion of THCs or CHCs offer inpatient services. Resource allocation such as health staff, beds, medical equipment, and building areas at THCs or CHCs, are closely related to the population they served and are regulated by the national government. Hospitals are equipped to provide more comprehensive medical services at the county and above level (city and province), referred to as “hospital care” in this study. Notably, the PHIs in China are neither the first point of contact (so-called gatekeepers) nor do they coordinate with hospital care.^10^ Two major health insurance programs in China—urban and rural residents basic medical insurance scheme and employees’ medical insurance scheme—encourage patients to obtain primary care first by setting a lower deductible and higher reimbursement rate at PHIs.^11^ However, since it was not mandatory, patients could choose to obtain hospital services directly. Further, they would be reimbursed by the corresponding medical insurance schemes.

 Due to low capacity,^12^ patients with minor diseases such as the common cold preferred visiting tertiary hospitals bypassing PHIs, leading to a massive waste of medical resources.^13^ Vertical integration was greatly emphasized along with the new round of healthcare reform initiated in 2009 to strengthen primary healthcare and establish a well-organized tiered healthcare delivery system. As mentioned above, vertical integration’s primary mechanism is to coordinate experienced professionals and sophisticated medical resources between the hospitals and the PHIs, through a series of organizational and management reforms and arrangements.^14,15^ Through this, it was expected that the competency of PHIs would be improved and they would have the ability to treat patients’ common diseases at the primary care level. Guided by national policy, local governments have piloted different vertically integrated care models. By learning from the intensity of integration as defined by Leutz,^16^ these models implemented in China could be further classified into two types based on the degree of integrative strategies related to administrative, organizational, and service delivery levels—“tight integration” and “loose collaboration.” With the reform deepening, it would be beneficial to understand the effects of vertical integration from the perspective of PHIs. Moreover, as one of the largest developing countries, China’s study could provide valuable evidence and greatly enrich international findings.

 To date, relatively more evidence on the impacts of vertical integration has been found from the perspective of diseases, particularly regarding specific conditions such as cancer^17^ and chronically illness,^18,19^ or the overall patients’ perceived quality of primary care.^20^ Rare evidence could be found to show the effects and differences across types of vertical integration on the PHIs in China. Although few studies have been published in Chinese journals regarding changes in service volume and patient referral under vertical integration,^21,22^ their reliability is severely limited by the cross-sectional study design and lack of quantitative data over a more extended period. Ample evidence with a longitudinal design was urgently needed to guide policy implementation in practice.

 This study aims to evaluate the effects of vertical integration reform on PHIs in China. Two research hypotheses are proposed: (1) the vertical integration policy will have a positive effect on the PHIs; and (2) regarding the intensity of vertical integration, tight integration will likely better influence the PHIs compared with loose collaboration.

## Methods

 The reform of vertical integration in China was guided by the government. Therefore, our study is a quasi-experimental study design instead of intervention design. In policy practice, treatments sometimes occur at different times. The processes that generate treatment variables naturally lead to variations in the timing.^23^ In this situation, the traditional standard difference-in-difference (DID) method with fixed treatment timing is limited. Time-varying DID is more appropriate for dealing with various treatment times. Therefore, considering the different integrated intervention times in our study, the time-varying DID method was adopted to estimate the effects of vertical integration on the PHIs.

###  Study Setting and Data Sources

 According to the representativeness of vertically integrated care models piloted, geographical distribution and data availability, five districts (counties) in Zhenjiang city (Jiangsu province, eastern China), Yichang city (Hubei province, central China), and Chengdu city (Sichuan province, western China) were purposively selected as study samples.

 Data were collected from health statistical data reporting system from 2009 to 2018 with local health departments’ permission in the sampled counties and districts. According to the health statistics department’s requirements in China, all PHIs and hospitals need to report and fill in the data about basic information and provision of health services in the uniform health statistical information system every year. It serves as the basis for the official health statistics yearbook. The data are filled at the unit of each health institution. PHIs and hospitals are required to first aggregate the number of outpatient visits and inpatient admissions data at the patient level recorded in their information system. Then, they need to upload them to the health statistical data reporting system. Local health departments are responsible for the management and use of the data. The indicators used in this study include human resources, number of beds at PHIs, inpatient and outpatient service volumes at both PHIs and hospitals, blood pressure control rate of hypertension (%), and blood glucose control rate of diabetes (%). Due to the limitation of availability, Wuhou District’s data only covered the period from 2012-2018. Finally, all 370 observations (PHI-year) in the sample counties and districts were included in this study. Since only a portion of PHIs provide inpatient services, the sample for inpatient-related outcome indicators was 280.

###  Measurement of Vertical Integration 

 Combining the integration domains defined by Kodner.^1,24^ and adaptation to the Chinese context, specific integrative strategies implemented in practice were divided into three aspects: administrative, organizational, and service delivery (Table 1). Although there were common strategies, their intensity varied noticeably across the types of integration.

**Table 1:** Attributes of Integrative Strategies for PHIs Under Two Types of Vertical Integration

**Main Integrative Strategies **	**Tight Integration **	**Loose Collaboration **
Administrative		
Contractual relationship	Yes. With detailed contents.	Yes. With nominal contents
Ownership of PHIs	The local government.	The local government.
Operation and management responsibilities over PHIs	The leading hospital.	PHIs.
Organizational		
Coordination office (to oversee collaboration and management)	2-3 persons or one department especially for the integration issue.	Part-time persons usually at service supervision department in hospitals.
New departments with high needs at PHIs	Establishing departments based on the characteristics at PHIs, such as rehabilitation, pediatric, or test/examination.	Rarely seen.
Sharing information system	Local uniform information system.	Local uniform information system.
Service delivery		
The channel for dual referral	Yes. In some cases, patients could pay for the bills for examination at PHIs but get served in hospitals based on the contract.	Yes. Upward referral for the majority.
Joint training program	Clear target on training PHI health professional, including frequency and number of hospital experts, priority clinical skills trained, etc. The program funding usually from the hospitals and local government, as common standard with 500 RMB/person/day.	Flexible, based on the local government’s requirement, or demanding PHIs or a personal contact between PHIs and hospitals.
Hospital specialist service	Hospital experts went to the PHIs to treat patients regularly, especially for the newly established department.	Flexible, based on the government requirements.

Abbreviation: PHIs, primary healthcare institutions

 The distribution of the PHIs along with the vertical integration reform is shown in Figure S1 of Supplementary file 1. Specifically, in the eastern area, Runzhou district and Jingkou district in Zhenjiang city started the reform in 2009 with an initial focus on coordination among public hospitals, while the clear emphasis on PHIs during vertical integration was initiated in 2012. All the CHCs in Runzhou district have been covered by tight integration while all the CHCs in Jingkou district have been covered by loose collaboration since 2012. In Zhijiang county (Yichang city), located in the middle area, two out of seven THCs have been tightly integrated with county level hospitals since 2013. The other five have been under loose collaboration with county level hospitals since 2016. In the western part, loose collaboration reform between hospitals and PHIs at both Wuhou district and Xinjin county in Chengdu city was initiated in 2016.

###  Outcome Measures 

 The goal of the vertical integration policy in China is to improve the competency and quality of PHIs and treat patients at the primary care level. To this end, we selected indicators in three dimensions to analyze their changes at PHIs: (1) Service volumes at PHIs, including the number of annual outpatient visits (No. visits) at PHIs and the number of annual inpatient admissions (No. admissions) at PHIs. (2) The patient flow between PHIs and hospitals, including the ratio of outpatient visits between PHIs and hospitals (ratio of visits = the number of outpatient visits at the PHI/the number of outpatient visits at the participating hospitals in the same integrated models, %), and the ratio of inpatient admissions between PHIs and hospitals (ratio of admissions = the number of inpatient admissions at the PHI/the number of inpatient admissions at the participating hospitals in the same integrated models, %). (3) Quality of primary care, given data availability, control rate of hypertension (%), and control rate of diabetes (%) were selected as surrogate quality indicators of primary care, as hypertension and diabetes are the two most common chronic conditions encountered in PHI settings.^10^ The PHIs in China are responsible for managing patients diagnosed with hypertension and diabetes (type II) in their jurisdiction, and usually cover several streets. For patients with hypertension and diabetes, PHIs are recommended to provide at least four follow-up visits for blood pressure measurements and to review drug utilization. Each managed patient has their health record to document the services provided. Therefore, the two indicators (control rate of hypertension and control rate of diabetes) could be calculated and were reported by PHIs to the health data reporting system. Specifically, the control rate of hypertension (%) = the number of hypertensive patients with systolic pressure <140 mm Hg and diastolic pressure <90 mm Hg (systolic pressure <150 mm Hg and diastolic pressure <90 mm Hg for hypertensive patients aged 65 and above) during the latest follow-up visit/the number of hypertensive patients under the management of PHIs*100%; control rate of diabetes (%) = the number of diabetic patients (type II) with fasting blood glucose <7 mmol/L during the latest follow-up visit, and the number of diabetic patients (type II) under the management of PHIs*100%.

###  Statistical Analysis 

 We used a quasi-experimental DIDs design (also known as an event study specification) to evaluate the effect of vertical integration on PHIs. We constructed a series of binary variables denoting leads and lags of vertical integration ranging from three or more years before vertical integration to four or more years after.


(1) $$\log\left(Y_{it}\right)=\propto +\sum_{2}^{3}\lambda_{j1}\times INTEGRATION_{it\left(t=k-j\right)}\times Type_{i}+\sum_{0}^{3}\lambda_{j1}\times INTEGRATION_{it\left(t=k+j\right)}\times Type_{i}+\beta X_{it}+\mu_{i}+\gamma_{t}+\delta_{mt}+\varepsilon_{it}$$

where we constructed seven dummy variables, *INTEGRATION*_it(t=k–j)_, for years 2-3 before adopting the integration, and *INTEGRATION*_it(t=k+j)_, for years 0-3 after adopting the integration. *k* is the time at which the integration is being switched on PHI*i*. Of these seven indicators, we noted that the first variable was equal to 1 in each year before the third year ahead of the implementation; the final variable was equal to 1 in each year, starting with the third year of implementation, and other years took on the value 1 only in the relevant year. Type_i_ took a value 0 for loose collaboration, and 1 for tight integration. *Y*_it_ represents the outcome variables in PHI (*i*) and year (*t*). Logarithmic transformations were performed using the No. of visits and No. of admissions to adjust for right-skewed data. Control variables, namely *X*_it_, included the number of medical staff and were also estimated in logs. All estimates included a vector of PHI’s individual fixed effects (*μ*_i_) that control for mean differences across PHIs, year dummies (*γ*_t_) that controlled for flexible year effects common to all hospitals, and city-specific time trend (*δ*_mt_) that relaxed the common trend assumption by allowing different cities to follow different trends. *ε*_it_ is the error term. Linear models with clustered standard errors at the PHI level were performed.

 Statistical significance was set at *P* < .05. Stata 16 for Windows (Stata Corp, College Station, TX, USA) was used for the statistical analysis.

## Results

###  Summary Statistics 

 The descriptive results for the PHIs are summarized in Table 2. It shows increases in the majority of outcome measures under both types of integration after reform, except for the ratio of visits and admissions ratio. The improvements at the PHIs under tight integration were more evident than those under the loose collaboration.

**Table 2:** Characteristics of Sample Primary Healthcare Institutions

**Variable**	**Loose Collaboration**				**Tight Integration**			
	**Pre**		**Post**		**Pre**		**Post**	
	**N**	**Mean (SD)**	**N**	**Mean (SD)**	**N**	**Mean (SD)**	**N**	**Mean (SD)**
No. of visits in PHI	180	71 819.3 (62 538.6)	107	117 047.5 (82 499.3)	22	99 934.7 (88 933.6)	61	147 142.3 (100 124.0)
No. of admissions in PHI	149	1971.9 (1393.4)	75	1537.8 (1132.9)	14	1073.6 (487.1)	42	2291.3 (2237.4)
No. of visits in hospital	173	2 854 930.0 (4 905 353.0)	110	3 502 019.0 (48 39514.0)	22	1 497 743.0 (997 631.2)	61	1 857 590.0 (778 790.4)
No. of admissions in hospital	173	118 305.6 (20 0974.6)	110	135 514.2 (197 544.3)	22	44 266.8 (20752.3)	61	73 607.4 (24 875.8)
Ratio of visits (%)	173	16.5 (17.2)	101	10.1 (9.7)	22	7.3 (3.1)	61	8.6 (4.5)
Ratio of admissions (%)	142	19.0 (26.2)	69	7.6 (9.7)	14	4.3 (2.9)	42	4.2 (3.8)
Control rate of hypertension (%)	112	65.3 (16.4)	115	69.9 (15.2)	2	52.2 (11.5)	58	62.4 (20.4)
Control rate of diabetes (%)	112	61.0 (14.4)	115	66.7 (14.7)	2	53.1 (17.3)	58	58.9 (19.0)
No. of medical staffs	180	19.0 (8.4)	124	22.8 (12.2)	22	20.9 (15.9)	61	20. 5(18.5)

Abbreviations: PHI, primary healthcare institution; SD, standard deviation. Note. N: PHI-year; No. visits: the number of annual outpatient visits; No. admissions: the number of annual inpatient admissions; Ratio of visits = the number of outpatient visits at the PHI/the number of outpatient visits at the participating hospitals in the same integrated models, %; Ratio of admissions = the number of inpatient admissions at the PHI/the number of inpatient admissions at the participating hospitals in the same integrated models, %; Control rate of hypertension (%) = the number of hypertensive patients with systolic pressure <140 mm Hg and diastolic pressure <90 mm Hg (systolic pressure <150 mm Hg and diastolic pressure <90 mm Hg for 65and elder hypertensive patients) during the latest follow-up visit/the number of hypertensive patients under the management of PHIs*100%; Control rate of diabetes (%) = the number of diabetic patients (type II) with fasting blood glucose <7 mmol/L during the latest follow-up visit/the number of diabetic patients (type II) under the management of PHIs*100%.

###  The Effects of Vertical Integration on the Related Indicators at PHIs

 Figure displays the point estimates and 95% confidence intervals (CIs) of the average effect of vertical integration on the related indicators at PHIs, calculated using equation (1). Each point represents the estimated effect of vertical integration on the specified outcome for a specified period (relative to the implementation of vertical integration). The corresponding regression results are listed in Table 3.

**Figure:**
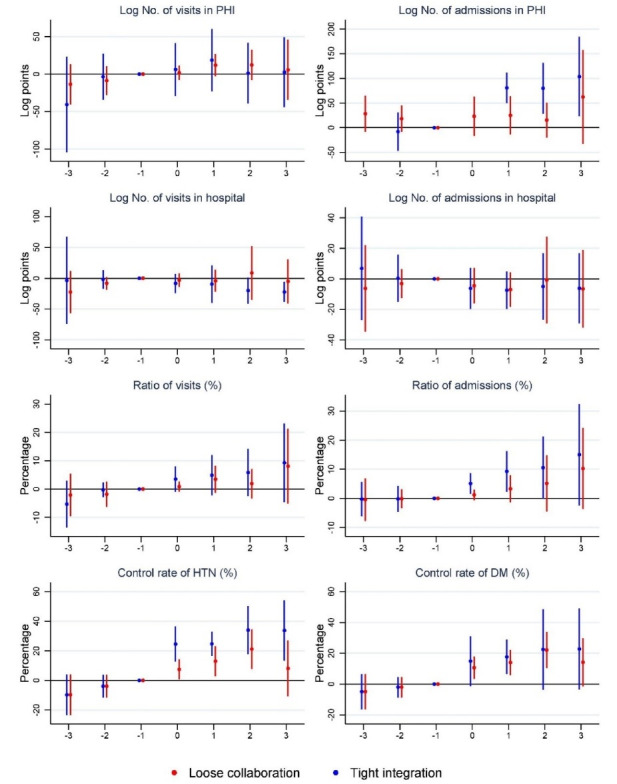
Event Study Estimates. Abbreviations: PHI, primary healthcare institution; DM, diabetes mellitus; HTN, Hypertension.

**Table 3:** Estimated Effects of Vertical Integration on the Related Indicators at Primary Healthcare Institutions

	**No. of Visits in PHI**		**No. of Admissions in PHI**	
	**Loose Collaboration**	**Tight Integration**	**Loose Collaboration**	**Tight Integration**
integration_t-3_	-0.136 (-0.404, 0.132)	-0.408 (-1.045, 0.230)	0.283 (-0.081, 0.648)	-2.375 (-5.230, 0.480)
integration_t-2_	-0.087 (-0.279, 0.105)	-0.035 (-0.342, 0.272)	0.183 (-0.081, 0.446)	-0.080 (-0.468, 0.308)
Integration_t0_	0.018 (-0.077, 0.112)	0.061 (-0.290, 0.412)	0.232 (-0.164, 0.628)	-0.043 (-0.802, 0.717)
integration_t+1_	0.120 (-0.027, 0.266)	0.187 (-0.228, 0.601)	0.251 (-0.134, 0.636)	0.808 (0.503, 1.113)^c^
integration_t+2_	0.122 (-0.081, 0.325)	0.013 (-0.391, 0.417)	0.155 (-0.197, 0.507)	0.800 (0.284, 1.317)^b^
integration_t+3_	0.057 (-0.345, 0.459)	0.026 (-0.439, 0.491)	0.624 (-0.327, 1.575)	1.036 (0.230, 1.843)^a^
	**No. of Visits in Hospital**		**No. of Admissions in Hospital**	
	**Loose Collaboration**	**Tight Integration**	**Loose Collaboration**	**Tight Integration**
integration_t-3_	-0.224 (-0.566, 0.117)	-0.036 (-0.742, 0.669)	-0.063 (-0.345, 0.220)	0.069 (-0.271, 0.408)
integration_t-2_	-0.082 (-0.184, 0.020)	-0.020 (-0.167, 0.128)	-0.031 (-0.126, 0.063)	0.004 (-0.150, 0.158)
Integration_t0_	-0.030 (-0.137, 0.077)	-0.084 (-0.240, 0.073)	-0.045 (-0.160, 0.071)	-0.062 (-0.196, 0.071)
integration_t+1_	-0.043 (-0.220, 0.134)	-0.095 (-0.398, 0.209)	-0.070 (-0.182, 0.042)	-0.074 (-0.197, 0.048)
integration_t+2_	0.086 (-0.349, 0.520)	-0.200 (-0.412, 0.012)	-0.007 (-0.291, 0.276)	-0.050 (-0.267, 0.167)
integration_t+3_	-0.052 (-0.410, 0.306)	-0.222 (-0.384, -0.060)	-0.066 (-0.319, 0.188)	-0.062 (-0.290, 0.166)
	**Ratio of Visits (%)**		**Ratio of Admissions (%)**	
	**Loose Collaboration**	**Tight Integration**	**Loose Collaboration**	**Tight Integration**
integration_t-3_	-2.110 (-9.568, 5.349)	-5.353 (-13.559, 2.854)	-0.481 (-7.788, 6.826)	-0.287 (-6.193, 5.620)
integration_t-2_	-1.852 (-6.321, 2.617)	-0.295 (-2.876, 2.286)	-0.138 (-3.386, 3.109)	-0.203 (-4.631, 4.226)
Integration_t0_	0.868 (-0.936, 2.672)	3.507 (-0.972, 7.986)	1.192 (-0.576, 2.960)	5.063 (1.508, 8.619)^b^
integration_t+1_	3.456 (-1.241, 8.154)	4.855 (-2.241, 11.950)	3.269 (-1.373, 7.911)	9.263 (2.257, 16.270)^a^
integration_t+2_	1.899 (-3.285, 7.083)	5.822 (-2.524, 14.168)	5.119 (-4.500, 14.738)	10.535 (-0.152, 21.223)^a^
integration_t+3_	8.069 (-5.123, 21.261)	9.258 (-4.620, 23.137)	10.247 (-3.643, 24.137)	14.992 (-2.398, 32.382)
	**Control Rate of Hypertension (%)**		**Control Rate of Diabetes (%)**	
	**Loose Collaboration**	**Tight Integration**	**Loose Collaboration**	**Tight Integration**
integration_t-3_	-9.705 (-23.301, 3.890)	-9.705 (-23.301, 3.890)	-4.913 (-16.314, 6.488)	-4.913 (-16.314, 6.488)
integration_t-2_	-3.895 (-11.537, 3.746)	-3.895 (-11.537, 3.746)	-2.000 (-8.622, 4.621)	-2.000 (-8.622, 4.621)
Integration_t0_	7.442 (0.680, 14.205)^a^	24.562 (12.717, 36.406)^c^	10.632 (3.445, 17.818)^b^	14.865 (-1.275, 31.006)^a^
integration_t+1_	12.970 (2.816, 23.124)^a^	24.699 (16.560, 32.838)^c^	14.018 (5.837, 22.199)^b^	17.655 (6.559, 28.752)^b^
integration_t+2_	21.189 (7.804, 34.573)^b^	33.992 (17.703, 50.280)^c^	22.088 (10.375, 33.802)^c^	22.456 (-3.547, 48.459)^a^
integration_t+3_	8.118 (-10.711, 26.947)	33.716 (13.324, 54.108)^b^	14.175 (-1.389, 29.739)	22.773 (-3.420, 48.966)

Abbreviation: PHI, primary healthcare institution. a^a^*P *< .05, b^b^*P *< .01, c^c^*P *< .001.

 The adoption leads’ coefficients are close to zero for all outcomes, demonstrating no evidence of differential trends in these outcomes. One year after tight integration, the number of admissions in PHIs increased substantially by 80 log points (124%=exp^0.808^-1), which increased the admissions ratio by 9.26 %. This value rapidly rose in the succeeding years, peaking at 104 log points (183%=exp^1.036^-1), with a 14.99 % increase. However, no significant increase in the number of admissions was observed after loose collaboration. For the control rate of hypertension and diabetes, while both loose and tight integration showed significant positive impacts, it increased more in the latter group. The most significant increases were 34.0% and 22.8% for the control rate of hypertension and diabetes, respectively, compared with the peak of 21.2% and 22.1% under the loose collaboration.

## Discussion

 Our study revealed that tight integration had significantly positive impacts on the absolute number of admissions in PHIs and the admissions ratio between PHIs and hospitals. Conversely, no significant effects were observed for these indicators at the PHIs after loose collaboration. Moreover, both types of integration had a significantly positive effect on quality of hypertension care and diabetes care. However, the improvement was more distinct for the PHIs under tight integration. The underlying reasons for the outcome variations are worth further discussions.

 First, there was a significant increase in inpatient admissions at PHIs under tight integration, which peaked at 183% improvement after intervention. Inpatient services are the priority and primary function of hospitals in China. Therefore, more efforts were devoted to strengthening the inpatient capacity of PHIs during integration. For example, under tight integration, common strategies included strong support for a newly developed department with high demand at the PHIs, and more quality medical resources, such as professionals, management staff, and advanced equipment at discount prices,^25^ devoted from the integrated hospitals to PHIs. Accompanied by specific training programs and full-time staff in charge of case management and care coordination, the newly established departments were widely explored, particularly for rehabilitation, testing, and examination. Further, they expanded the professional domains of PHIs to attract and retain more inpatients, which finally increased primary care utilization.^26^ These strategies, which are usually detailed in the form of contracts, were implemented in a planned and regular way under tight integration.^27^ However, they were rarely observed for the PHIs under loose collaboration or those without integration. Instead, providing professional guidance from the hospitals was more occasionally or temporarily based on PHIs’ demands under the loose collaboration.

 Second, the changes in patient flow, denoted by a significant increase in the ratio of admissions between the PHIs and the hospitals under tight integration, demonstrated a good sign, indicating that the increment of PHI inpatient care utilization exceeded the increase of hospitals during the study period. Such changes are worth more attention as they satisfy the original intention of vertical integration policy reform in China, as it retains more patients at the primary care level. Similarly, by using health insurance data from 2012 to 2016 of four counties in Anhui province, Cheng et al in 2018^21^ found the positive effects of tight integration on inpatient admissions and dual referral by descriptive analysis. In addition to the reasons leading to the increase in inpatient service volume described in the first aspect, the dual referral between the integrated PHIs and hospitals also contributed to the increase. Under tight integration, the leading hospitals usually took over the operation and management responsibilities of PHIs at the administrative level and set up special offices with full-time staff in charge of care coordination at the organizational level, which stimulated the hospitals and the PHIs to act as a whole. Therefore, hospitals were more prone to refer suitable patients to the PHIs. All these strategies probably stimulated the inpatients flow to the PHIs under tight integration. Additionally, we used total inpatient admissions of participating hospitals in the same integrated models to calculate the ratio indicators determined by the characteristics of different integrated care models. It should be noted that the positive effects on the ratio of inpatient service volumes between PHIs and hospitals may be underestimated due to the dilution of the denominators of the ratio values. All these strategies probably stimulated the inpatients flow to the PHIs under tight integration.

 Additionally, we used total inpatient admissions of participating hospitals in the same integrated models to calculate the ratio indicators determined by the characteristics of different integrated care models. It should be noted that the positive effects on the ratio of inpatient service volumes between PHIs and hospitals may be underestimated due to the dilution of the denominators of the ratio values.

 Third, it was meaningful to find, in this study, that both types of integration significantly improved the quality of care related to hypertension and diabetes, particularly in tight integration. Better quality of treatment for chronic diseases is usually supported by better clinical skills.^19^ After vertical integration, the leading hospitals initiated clinical skills training of PHI health professionals for common diseases, particularly chronic ones. Typical training schedules included a comprehensive study of evidence-based practice standards and protocols for diseases such as hypertension, diabetes, and stroke, and person-to-person teaching at hospitals and PHIs. The improvement of professional skills for PHI professionals’ chronic diseases could be one of the most important reasons for better quality of care. Although both types of integration shared some common training strategies, they were more regular and more intensive under tight integration, which could explain the higher improvement in the quality of hypertension and diabetes care under this integration.^10^ Further, the quality improvement of care for hypertension and diabetes was also possibly affected by changes in patient composition. Due to free choices of medical institutions, it was more likely for hypertensive and diabetic patients with lighter symptoms, and for those patients with better health outcomes to visit PHIs instead of hospitals.

 Lastly, it is worth noting the context of implementing vertical integration in China. There were huge discrepancies between PHIs and hospitals regarding both capacity and medical resources.^10,12^ Improving the competency of PHIs was one of the most critical goals expected from vertical integration reform. Consequently, in the present integrated care pilots, participating hospitals have played a vital role, while the PHIs have been receivers of all integrative strategies. During the initial assessment in our study, positive impacts on PHIs were observed, as illustrated above. However, the rationale for the hospital-leading role in integrated care pilots must be considered carefully in the future and in the context of other countries, considering the different functions of PHIs and hospitals. For example, evidence from accountable care organizations (ACOs) in America showed that physician-group ACOs were associated with Medicare savings, while hospital-integrated ACOs did not produce savings.^28^ Additionally, incentives, such as shared-savings bonuses rewarded to ACOs^28-30^ were lacking during ongoing reform in China; they mainly relied on local governments’ administrative power. Recently, some counties in China initiated bundled payment reform with the aim of establishing long-term incentives between PHIs and hospitals.^26,31^ However, the effect of these initiatives need a longer period to be examined.

 This study has several limitations. First, local pilots of integrated care models vary across counties with special characteristics. We selected the samples considering the representative, geographical, and socioeconomic status and controlled possible variables. Nonetheless, it was not possible for us to obtain all districts data covered by each integration type under quasi-experimental design. Therefore, the data restriction may lower the power of our analysis and the estimates may not reflect the effects of policy changes among all districts exposed to policy changes. Cautious attitude needs to be kept towards our conclusions. Second, we used quality of care related to hypertension and diabetes (two typical chronic diseases encountered in primary care settings) to surrogate quality of care provided by PHIs based on the data availability. These were more likely to be affected by the vertical intervention and may overestimate tight integration’s positive effects. Additionally, due to policy restrictions in Wuhou District, the data before 2012 were not available, resulting in some bias. Third, although the estimates used in our study is widely applied in empirical economics,^32-35^ it is still worth paying attention to possible bias resulted by logarithmic transform of dependent variables. Puhani^36^ discussed that the treatment effect for nonlinear models with limited dependent variables (such as probit, logit, or tobit models) is the cross difference of the observed outcome minus the cross difference of the potential non-treatment outcome. The CI of this treatment effect may be obtained using a bootstrap approach. It enlightens future study concerning policy effects by DID analysis to try this solution and discuss the feasibility in a log-linear model. However, despite these limitations and to the best of our knowledge, this is the first long-term study to examine the quantitative effects of vertical integration on the service volume, patient flow, and quality of primary care at PHIs by DID design. In this regard, it could greatly enrich the evidence on the academic side; more importantly, it provided valuable guidance for further integration reform in China and other countries in practice.

###  Policy Implications 

 Addressing fragmentation and providing integrated and continuous healthcare remains challenging, particularly in developing countries.^8^ Based on the results and discussion above, three policy implications are proposed to enlighten the countries on strengthening primary healthcare-based integrated health systems. First, our findings are more prone to support tight integration between PHIs and hospitals when designing vertical integration reform due to the significant positive effects of inpatient service volume, inpatient flow, and quality of care. Second, the positive effects obtained were closely related to the specific strategies implemented and the vertical integration reform. Priority strategies are therefore suggested, including strengthening the leading hospitals’ operation and management responsibilities over PHIs at the administrative level, establishing new departments with high needs, such as rehabilitation beds at PHIs and coordination offices with full-time staff at the organizational level, and designing professional training programs consistent with the PHI functions. Further, training must be provided in a planned and regular way at the service delivery level, which were regarded as possible reasons for the positive effects. Lastly, the development of an integrated care model is strongly contextually bound. The success of vertical integration must be closely connected with the health system’s structure, the basis of primary care, and the characteristics of the population it aims to serve.

## Conclusion

 Our study explored vertical integration piloting experiences and their effects on PHIs in China. The findings are not only academically valuable, but it also provides empirical support for policy-makers to strengthen primary healthcare through vertical integration reform. Given the positive effects on inpatient service volume, inpatient flow, and quality of hypertension and diabetes care at the integrated PHIs, our study suggests that vertical integration (especially tight integration) in China significantly contributed to strengthening primary healthcare, providing empirical evidence for other countries aiming to establish integrated primary healthcare-based health systems.

## Acknowledgements

 The authors are grateful to the staff in the local health bureaus and THCs in Zhenjiang city of Jiangsu province, Yichang city of Hubei province, and Chengdu city of Sichuan province for their help in data collection and relevant documents’ assistance. Faculty members and postgraduates from Institute of Medical Information and Library are appreciated for their work in field survey.

## Ethical issues

 This study was approved by the Institute of Medical Information & Library Human Research Ethics Committee (HREC) (Ref. No. IMICAMS/01/19/HREC).

## Competing interests

 Authors declare that they have no competing interests.

## Authors’ contributions

 SY conceptualized study design, analyzed data and drafted the manuscript; FF provided advice on the analysis framework and critical revision of the manuscript; DZ conducted data analysis, and provided critical revision of the manuscript; all authors contributed to the data interpretation.

## Funding

 This work was supported by the National Office for Philosophy and Social Sciences [16CGL067].

## Supplementary files

Supplementary file 1 contains FIgure S1.
