# Supplementary material for: Effects of Vertical Integration Reform on Primary Healthcare Institutions in China: Evidence From a Longitudinal Study
Source: Int J Health Policy Manag. 2021 Aug 21;11(9):1835–43. doi: 10.34172/ijhpm.2021.93 (PMC9808208; doi:10.34172/ijhpm.2021.93)
Supplement: Supplementary file 1 — contains FIgure S1. [file ijhpm-11-1835-s001.pdf]

Article title: Effects of Vertical Integration Reform on Primary Healthcare Institutions in China: Evidence from a Longitudinal Study

Journal name: International Journal of Health Policy and Management (IJHPM)

Authors' information: Shasha Yuan[1], Fengmei Fan[2]*, Dawei Zhu[3]*

[1]Institute of Medical Information & Library, Chinese Academy of Medical Sciences & Peking Union Medical College, Beijing, China.

[2]Beijing Huilongguan Hospital, Peking University Huilongguan Clinical Medical School, Beijing, China.

[3]China Center for Health Development Studies, Peking University, Beijing, China.

(*Corresponding authors: fanfengmei@live.com & zhu_dawei@163.com)

Supplementary file 1.

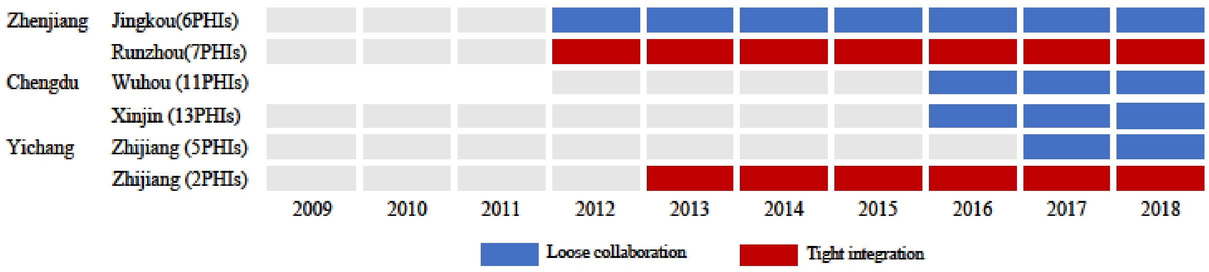

Figure S1. The distribution of primary healthcare institutions under tight integration and loose collaboration in sample areas
